# Characteristics of gram-negative urinary tract infections caused by extended spectrum beta lactamases: pivmecillinam as a treatment option within South Dublin, Ireland

**DOI:** 10.1186/s12879-016-1797-3

**Published:** 2016-11-03

**Authors:** Fardod O’Kelly, Siobhan Kavanagh, Rustom Manecksha, John Thornhill, Jérôme P. Fennell

**Affiliations:** 1 Department of Clinical Microbiology, AMNCH, Tallaght Hospital, Dublin 24, Ireland; 2 Department of Urological Surgery, AMNCH, Tallaght Hospital, Dublin 24, Ireland

**Keywords:** Extended-spectrum beta lactamase, Pivmecillinam, Antibiotic resistance, Urinary tract infection, Mean inhibitory concentration, *Escherichia coli*

## Abstract

**Background:**

The prevalence of urinary tract infections (UTIs) caused by extended-spectrum β-lactamase (ESBL)-producing Enterobacteriaceae is increasing and the therapeutic options are limited, especially in primary care. Recent indications have suggested pivmecillinam to be a suitable option. This pilot study aimed to assess the viability of pivmecillinam as a therapeutic option in a Dublin cohort ﻿of mixed community and healthcare origin﻿.

**Methods:**

A prospective measurement of mean and fractional inhibitory concentrations of antibiotic use in 95 patients diagnosed with UTI caused by ESBL-producing Enterobacteriaceae was carried out. 36 % patients were from general practice, 40 % were admitted to hospital within south Dublin, and 25 % samples arose from nursing homes. EUCAST breakpoints were used to determine if an isolate was sensitive or resistant to antibiotic agents.

**Results:**

Sixty-nine percent of patients (*N* = 66) with urinary ESBL isolates were female. The mean age of females was 66 years compared with a mean age of 74 years for males. Thirty-six percent of isolates originated from primary care, hospital inpatients (26 %), and nursing homes (24 %). The vast majority of ESBL isolates were *E. coli* (80 %). The E tests for mecillinam and co-amoxiclav had concentration ranges from 0.16 mg/L up to 256 mg/L. The mean inhibitory concentration (MIC) of mecillinam ranged from 0.25 to 256 mg/L, while co-amoxiclav MICs ranged from 6 to 256 mg/L. The percentage of isolates resistant to mecillinam and co-amoxiclav was found to be 5.26 and 94.74 % respectively.

**Conclusions:**

This is the first study exploring the use of pivmecillinam in an Irish cohort and has demonstrated that its use in conjunction with or without co-amoxiclav is an appropriate and useful treatment for urinary tract infections caused by ESBL-producing organisms.

## Background

Microorganisms constantly evolve resistance to antimicrobials, rendering current agents ineffective. This is compounded by the reality that there are few new antimicrobials in development. Extended-spectrum beta lactamase (ESBL) producing organisms are one of the resistance types of most concern. ESBLs were first recognized in the 1980s due to point mutations of Temoneira (TEM) and Sulphydryl Variable (SHV) broad-spectrum enzymes genes. A common causes of hospital-acquired infections especially in the intensive care unit, ESBLs also commonly carry resistance to other antimicrobials such as quinolones, cotrimoxazole and the aminoglycosides. This further limits treatment options. Laboratory diagnosis of ESBLs is complex and normally performed by screening and phenotypic tests [1].

Traditionally ESBL producing bacteria were associated with nosocomial infection, but they are now widespread in the community. In the UK, community ESBLs were mostly isolated during urinary infections the elderly, who had recent hospitalisation [2]. In contrast, an Irish study found that 42 % of community isolates that were ESBL producing, were from individuals not in long term care or hospitalised in the previous year [3]. Risk factors for acquiring community associated ESBL infections include recurrent UTI, previous antibiotic usage, diabetes and prior instrumentation to urinary tract [1, 4].

ESBLs were first detected in Western Europe, where β-lactam antibiotics were first used, with prevalence varying between countries. In 2001, the Netherlands had a low percentage of ESBL producing Enterobacteriaceae with only 1 % of *E. coli* and *K. pneumoniae* being ESBL positive. In contrast 40 % of France’s *K. pneumoniae* were ceftazidime resistant. ESBL producers are normally multiple drug resistant and have become an important mechanism of β-lactam resistance in community uropathogens [5]. Production of β-lactamase is the most common resistance mechanism of bacteria to β-lactam antibiotics [6]. *E. coli* resistance is mostly due to production of β-lactamases, which hydrolyze the beta-lactam ring of beta-lactam antibiotics such as penicillin. Resistance to ampicillin and amoxicillin is normally due to plasmid-coded β-lactamases the majority of which is the TEM type [7]. ESBLs have resistance to β-lactams, ampicillin, amoxicillin and third generation cephalosporins. The ESBL carrying plasmid often carries other resistance genes as well, e.g. resistance genes to quinolones and aminoglycosides. When this occurs usage of any of the classes of antimicrobials that the plasmid encodes resistance to will select for this multiple resistant isolate. The first ESBLs in *E. coli* were variants of the TEM or SHV β-lactamases, which could hydrolyze cefotaxime, ceftriaxone and ceftazidime, however the CTX-M-type *bla* gene has now become the commonest type. The CTX-M enzymes also appear to have a greater ability to spread and cause outbreaks [2, 8, 9]. CLSI recommendations state to only check for ESBLs in *E. coli, Klebsiella pneumoniae, K. oxytoca* and *Proteus mirabilis*, but all Gram-negative bacteria can be ESBL positive. In 2006, two *E. coli* ESBL isolates were associated with UTIs from two residents in an Irish nursing home. On review, five more patients in that nursing home were found to be ESBL positive [10]. This is a typical example of﻿ the transmission of antibiotic resistant bacteria in a vulnerable group of patients where long-term isolation is not viable and there is a  need to control the spread of these organisms. A more recent study surveyed an Irish nursing home and found over 55 % of residents were colonized by ESBL producers [11]. In Ireland in 2015, 10.6 % of invasive *E. coli* and 13.3 % of invasive *K. pneumoniae* isolates were found to be ESBL Positive, the highest annual percentage to date [12].

There is a lack of effective therapeutic options to combat ESBLs. Carbapenems, often regarded the antibiotic of choice, should be used when there are no other options available but their use inevitably leads to the emergence of carbapenem-resistant enterobacteriaciae (CRE). Fluoroquinolones, can be effective against ESBLs, but are not recommended for routine use due to resistance rates. Aminoglycosides, also effective, should not be used for monotherapy in serious infections, as they are bacteriostatic. Colistin should be used with caution, as it is a broad-spectrum agent. The potential nephrotoxicity of these agents is another concern in this setting and is another reason to limit their use. Tigecycline demonstrates good in vitro activity against ESBLs but the FDA has warned against its use due to the increased mortality in Tigecycline-treated patients, as well as its relative inefficacy in pneumonia and bacteraemia, as well as limited GU tract concentrations [13, 14]. Fosfomycin, an old broad-spectrum antibiotic, has been re-evaluated for the treatment of UTIs due to multidrug resistant organisms. It is only licensed for lower uncomplicated UTIs and may develop resistance [15].

Pivmecillinam, a β-lactam antibiotic, the prodrug of mecillinam, is hydrolyzed to the active agent mecillinam [16]. Mecillinam must be administered parentally but oral pivmecillinam is available. Its mode of action is to bind to penicillin-binding protein 2 in Enterobacteriaceae and inhibit bacterial cell wall synthesis [17]. It has high activity against many Gram-negative bacteria such as *E. coli, Klebsiella sp.,* *Salmonella sp.* and *Enterobacter sp.* and has limited activity against some Gram positives. The use of pivmecillinam for more than 20 years in Nordic countries confirms its efficiency and safety in treating UTIs. Its oral bioavailability makes it an attractive option without requiring hospital admission for intravenous treatment [16, 18].

Antibiotic resistance is a concerning public health problem that increases morbidity and mortality. Novel drug development is time-consuming, but re-evaluating antibiotics already licensed is more time-effective [17]. Combination therapy is of interest as multi drug resistant microorganisms may require more than one antibiotic to treat successfully [19]. Unfortunately, there are limited oral antibiotics available for complicated UTIs caused by ESBL and AmpC producing bacteria [15].

This clinical study examines the antimicrobial susceptibility of 95 ESBL producing isolates to pivmecillinam and co-amoxiclav in a tertiary referral center, to determine if our catchment population could benefit with combination treatment of ESBL urinary tract infection.

## Methods

This study received approval from the hospital ethics committee and the research carried out was also in compliance with the Helsinki Declaration. Clinico-demographic data was collated on 95 patients that had tested positive for ESBL urinary infection at the department of Microbiology, Tallaght hospital (2012–2013). Isolates had previously been confirmed as ESBLs by MASTDISCS ID AmpC and ESBL inhibitors and MASTDISCS cefepime ESBL ID for the Vitek 2. E-tests were performed on ESBLs that were initially stored on beads in an −18 °C freezer, and then plated on MacConkey agar and incubated at 37 °C for 24 h. All isolates were then subcultured onto nutrient agar slopes. One mecillinam and one co-amoxiclav E test were then each applied with forceps onto the plate. The elliptical zones of inhibition were read to determine the MIC of *E. coli* according to the manufacturer instructions. The MIC was read as the point where the ellipse intersected the E test strip. If the intersect was different on both sides the greater value was taken as the MIC.

A checkerboard method was also used to assess the mean inhibitory concentrations (MIC) of each positive ESBL sample by measuring the spectrophotometric absorbance of each well 16 to 24 h following inoculation using an ELX800 universal micro plate reader at a wavelength of 630 nm. The percentage of growth was calculated on the basis of colour absorbance using (OD630 of wells that contained the drug/OD630 of the drug-free well). The MICs of the drugs alone and of in combination were determined as the lowest drug concentrations showing <10 % of the growth of an untreated control [20, 21].

For each combination of antibiotics the fractional inhibitory concentration was calculated which is a predictor of synergy [22]. The fractional inhibitory concentration (FIC) was used to evaluate the effectiveness of the combinations. The formulas used were FIC of drug A (mecillinam) = MIC drug A in combination/ MIC drug A alone; FIC of drug B (co-amoxiclav) = MIC drug B in combination/ MIC drug B alone and FIC index = FIC drug A + FIC drug B. Synergy was defined as FIC index of ≤0.5, indifference of FIC as >0.5 but of ≤4. Antagonism was defined as FIC index of >4. All experiments were performed in triplicate.

**Fig. 1:**
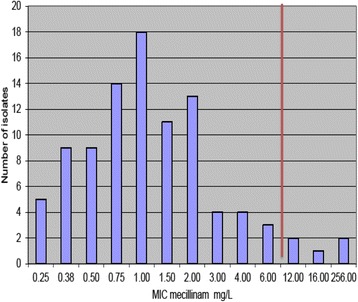
Distribution of the mean inhibitory concentration (MIC) of 95 Extended spectrum beta-lactamase (ESBL) producing isolates. EUCAST break-point of 8 mg/L demonstrated by *red vertical line*

**Fig. 2:**
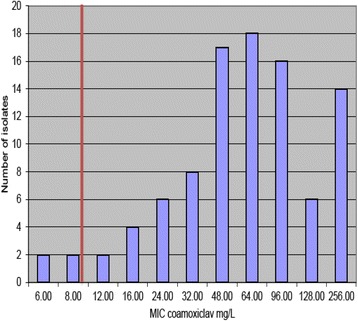
Distribution of the mean inhibitory concentration (MIC) of 95 Extended spectrum beta-lactamase (ESBL) producing isolates. EUCAST break-point of 8 mg/L demonstrated by *red vertical line*

EUCAST (European Committee on Antimicrobial Susceptibility Testing) breakpoints for Enterobacteriaceae for both mecillinam and co-amoxiclav were given as ≤8 mg/L as sensitive and ≥8 mg/L as resistant. However the British Society of Antimicrobial Chemotherapy states that an MIC of ≥32 mg/L for co-amoxiclav is suitable for UTIs but not systemic infections, due to the activity of clavulanate alone. Mecillinam concentrations in urine after 400 mg taken orally has been reported to be above 100 mg/L after 6-h. Co-amoxiclav has also been found to have levels above the MIC in human serum during the first 6 h [23]. Therefore the EUCAST breakpoints were used to determine if the isolate was sensitive or resistant to both antibiotic agents. The red line in Figs. 1 and 2 illustrate the EUCAST breakpoint of 8 mg/L.

## Results

Sixty-nine percent of patients (*N* = 66) with urinary ESBL isolates were female. The mean age of females was 66 years compared with a mean age of 74 years for males. Thirty-six percent of isolates originated from primary care, hospital inpatients (26 %), and nursing homes (24 %). The vast majority of ESBL isolates were *E. coli* (80 %) (Tables 1 and 2).

**Table 1:** Clinico-demographic profile of patients isolated with extended-spectrum beta lactamase producing urinary infections

Average age of patients (yrs)	%	N =
Female	66.25	n.a.
Male	74.65	n.a.
Source of Isolation of UTI		
Emergency Department	14	13
General Practice	36	34
Inpatient Wards	26	25
Nursing Home	24	23
Species Isolated		
*Klebsiella pneumoniae*	20	19
*Escherichia coli*	80	76

**Table 2:** Fractional inhibitory concentration (FIC) of the five extended-spectrum beta lactamase producing isolates which were resistant to mecillinam monotherapy

Isolate	FIC
842	Synergy
513	Synergy
779	Indifference
349	Indifference
031	Indifference

The E tests for mecillinam and co-amoxiclav had concentration ranges from 0.16 mg/L up to 256 mg/L. The mean inhibitory concentration (MIC) of mecillinam ranged from 0.25 to 256 mg/L, while co-amoxiclav MICs ranged from 6 to 256 mg/L. The MICs of mecillinam and co-amoxiclav were recorded and the total counts of the MIC were calculated. Distribution charts were constructed from this data (Figs. 1 and 2).

The percentage of isolates resistant to mecillinam and co-amoxiclav was found to be 5.26 % and 94.74 % respectively [24]. Four of five isolates were *E. coli* and were resistant to co-amoxiclav. Isolate number 513 was a *Klebsiella* species. These isolates were tested using the checkerboard technique for synergy between co-amoxiclav and mecillinam. The MIC was reduced significantly in isolates 842 and 513 in comparison to the E tests where only one drug was used. In the checkerboard method isolate 842 was inhibited with 24ug/ml of co-amoxiclav in combination with 12ug/ml of mecillinam. In the E test isolate 842 was inhibited by 12ug/ml alone but was not inhibited by co-amoxiclav until a concentration of 48 mg/L was used. Isolate 513 was inhibited at 24ug/ml of co-amoxiclav in combination with 64ug/ml mecillinam in the 96 well plate. In the E test isolate 513 was not inhibited by co-amoxiclav even at 256 mg/L. Isolates 779 and 031 did not achieve levels of 10 % of the growth of the control. Therefore the MIC for either or both drugs in combination was higher than 256ug/ml. In contrast the E test results showed isolate 779 was inhibited by co-amoxiclav at 128 mg/L but the results agreed with the checkerboard for mecillinam (>256 mg/L). The E test showed that 031 was inhibited by 12 mg/L of mecillinam but was not inhibited by co-amoxiclav even at 256 mg/L. The FIC is correct in that no positive difference occurred when the two antibiotics were used in combination. Isolate 349 was inhibited by 128 mg/L of mecillinam alone and also in combination with co-amoxiclav (between 6-256 ug/ml) in the 96 well plate. The E test results showed mecillinam did not inhibit the isolate at even 256 mg/L but co-amoxiclav inhibited 349 at 128 mg/L alone.

## Discussion

The increasing prevalence of clinically significant antibiotic resistant bacteria, especially for those resistant to multiple classes of antibiotics, makes appropriate antimicrobial treatment challenging [25, 26]. Many pathogens resistant to first line agents, then require broader spectrum, more expensive agents with less favorable safety profiles, which in turn accelerates the generation of multi drug resistant (MDR) pathogens, where no agents may be available to treat infections caused by these microorganisms. As the ESBL resistance mechanism is commonly expressed by plasmid encoded B lactamases, resistance can be easily transferred between other bacterial species by horizontal gene transfer. These plasmids frequently carry genes encoding for resistance to other antibiotic classes. Unlike methicillin resistant *Staphylococcus aureus*, the resistant Gram-negative bacteria commonly colonise the bowel and there is no available decolonization procedure. Long-term colonization by these bacteria ensures these patients remain a potential source of transmission for many years, with potentially indefinite requirement for isolation and contact precautions [27, 28].

There is an urgent need for more research into new agents that are effective against ESBLs and other resistant bacteria. With the increase in multidrug and carbapenem-resistant microorganisms, there is a need to minimize carbapenem use and they should not be first-line choice for treating ESBLs [29]. Europe-wide antimicrobial drug resistance rates remain high. A Greek study carried out in 2007 demonstrated CRE in 70 % of hospitals. Pan-drug resistant *K. pneumoniae* have also been reported in the same population. The use of mecillinam for UTIs is promising as pivmecillinam achieves high levels in urine, has a low resistance profile and inhibited 94.74 % of the ESBLs in this study. However, a broader clinical evaluation in an Irish patient population is required before clinical use can be advocated. Mecillinam like other penicillins, has low toxicity like other penicillins and is also safe to use for the treatment of UTIs in pregnancy [30]. Mecillinam is poorly absorbed from the gastrointestinal tract but pivmecillinam is well absorbed. Pivmecillinam reaches serum concentrations of 3ug/ml 1.5 h after 200 mg taken orally [31].

Inappropriate use of broad-spectrum antibiotics such as carbapenems adds to the problem of antimicrobial resistance as well as being responsible for a rise in *C. difficile* infections and healthcare costs. This study showed that mecillinam alone has good in vitro activity against clinical isolates of ESBL producing *E. coli* and *Klebsiella*.

Carbapenems are often used to treat UTIs caused by ESBLs; however this has been shown to lead to further resistance and will exacerbate the CRE problem. Invasive CRE infections have limited therapeutic options with mortality rates in excess of 40 % [32]. Pivmecillinam, widely used in Scandinavian countries for treating uncomplicated UTIs has a good safety profile [33]. Side effects of mecillinam include rash, nausea and vomiting. Surveillance in countries that strictly limit pivmecillinam for acute uncomplicated UTIs has shown low resistance in the community despite widespread use for more than 20 years [16]. There are reports of mecillinam resistance in *E. coli*; one mechanism is increased levels of ppGpp, a nucleotide effector [34]. Other studies have shown a low probability of future clonal spread of mecillinam resistance due to little association of resistance due to specific clonal groups. Other authors found that mecillinam resistance is not associated with ESBL production [35, 36].

In this study only 5.36 % of ESBLs that were E tested were resistant to mecillinam according to the EUCAST breakpoints with only two isolates having an MIC of >256 mg/L. This may be due the fact that mecillinam is only slightly affected by TEM and SHV1, which are the most frequent beta lactamases found in *E. coli*. In a French study, clinical efficiency was high with pivmecillinam and independent of MIC, which suggests susceptibility testing for UTIs caused by *E. coli* is not required with pivmecillinam, and could possibly be given empirically [17]. The results of the E tests with coamoxiclav show that only 2.11 % of isolates were susceptible to the drug according to the CLSI breakpoints, however according to the BSAC breakpoints, 25 % would be effective treatment of UTIs. Resistance to coamoxiclav is common and in a Spanish study 30.6 % of its ESBLs population were non-susceptible. The principle risk factor in that study was previous use of coamoxiclav, confirming that increased consumption leads to increased resistance. Resistance mechanisms of *E. coli* to coamoxiclav include B-lactamase overproduction, AmpC cephalosporinase hyperproduction and inhibitor-resistant penicillinases [37]. Resistance is acquired by clonal and non-clonal spread, dissemination of mobile elements with different *bla* genes and eventual mutations in individuals organisms [38].

Coamoxiclav alone was ineffective against 97.89 % of the isolates tested and should not be used alone for treatment of ESBL positive UTIs, however, the combination of coamoxiclav and mecillinam decreased the MIC significantly compared to mecillinam or coamoxiclav alone in three isolates. However two isolates had higher MICs with the combination of the two antibiotics compared to treatment with one antibiotic. For isolate 779 the coamoxiclav MIC increased from 128 mg/L alone to >256ug/ml when combined with mecillinam. Similarly but more worryingly isolate 031 was inhibited by 12 mg/L of mecillinam alone in the E test but was not inhibited by 256ug/ml in combination with coamoxiclav. The results for the mecillinam and coamoxiclav only in the 96 well plate differed to the results of the drugs alone in the E test. It is conceivable that some of these infections could represent identical bacterial strains, however, patients were disparate in time and place, and therefore it is likely that they represent different strains. This demonstrates that the combination of coamoxiclav and pivmecillinam on different species that produce ESBLs could be beneficial. However, further studies using mecillinam monotherapy or in combination with clavulanic acid, would benefit with a larger sample size to improve statistical power.

## Conclusion

Pivmecillinam alone appears to be a suitable treatment for UTIs due to ESBL producing Enterobacteriaceae especially lower UTIs. Resistance to this agent has been low even though it has been used for treating UTIs in Nordic countries for more than 20 years. In the future pivmecillinam in combination with coamoxiclav, would seem to be an appropriate treatment alternative for Irish ESBL infections and will also help reduce carbapenem usage, which in turn should help reduce the generation of CRE producing organisms. However for combination therapy further clinical evaluation would be required before clinical use could be advocated.

## Abbreviations

CRE: Carbapenem Resistant Enterobacteriaceae
ESBL: Extended spectrum beta lactamase
EUCAST: European Committee on Antimicrobial Susceptibility Testing
FIC: Fractional inhibitory concentration
MIC: Mean inhibitory concentration
PBP: Penicillin binding protein
SHV: Sulfhydryl variable beta-lactamases
TEM: Temoneira beta-lactamases
UTI: Urinary tract infection
